# Factors Associated With Failure of Non-invasive Ventilation in Preterm Neonates Requiring Initial Respiratory Support

**DOI:** 10.7759/cureus.53879

**Published:** 2024-02-08

**Authors:** Abhishek Nath, Sushil Srivastava, Ravi Sachan, Dheeraj Shah

**Affiliations:** 1 Pediatrics, University College of Medical Sciences, New Delhi, IND

**Keywords:** noninvasive ventilation (niv), nasal intermittent positive pressure ventilation (nippv), continuous positive airway pressure (cpap), failure factors, surfactant, fio2, preterm neonates

## Abstract

Background: Non-invasive ventilation (NIV) modalities minimize the requirement for invasive mechanical ventilation (IMV) in preterm neonates, therefore improving neonatal outcomes, as IMV is linked to increased complications. However, NIV has demonstrated an elevated likelihood of failure, for which various studies have been done, but very little research is available addressing the factors that are responsible for NIV failure in resource-limited areas of developing nations. Understanding the underlying factors and their association with NIV failure in very and moderately preterm neonates at a tertiary care hospital would be important in devising targeted strategies to increase NIV success and newborn outcomes.

Objective: To compare the following factors in neonates of 28-34 weeks gestational age with or without failure of NIV: fraction of inspired oxygen (FiO_2_) at the time of initiating NIV, time at surfactant administration, respiratory distress syndrome presence, antenatal steroid use, time taken for post-surfactant administration stabilization, gestational age, development of bronchopulmonary dysplasia, and average weight gained or lost.

Study design and participants: This was a longitudinal observational study. One hundred two preterm neonates with a gestational age of 28-34 weeks in the neonatal intensive care unit (NICU) requiring NIV support within 24 hours of admission.

Methods: Eligible newborns were re-evaluated at 72 hours after commencing NIV. Outcome was evaluated as success (no NIV or NIV with positive end-expiratory pressure (PEEP)<8 cm H_2_O and FiO_2_<0.7) or failure (NIV with PEEP≥8 cm H_2_O or FiO_2_≥0.7, intubation, or death). It was compared with regard to many parameters.

Results: About 40 (39%) study participants reported NIV failure within 72 hours of initiating NIV. In the NIV failure group, male babies constituted 75% (P = 0.027), the median gestational age (IQR) was 29 (29-31) weeks (P = 0.015), the median birth weight (IQR) was 1088 (960-1293.5) grams (P = 0.003), and the median weight gain or loss (IQR) was a loss of 21 (-70.5 to 11.75) grams (P<0.001). Vaginal birth comprised 67.5% of the NIV failure group, showing greater failure rates than births out of lower segment cesarean section (LSCS) (P = 0.003)

Conclusion: NIV failure showed a significant association with lesser gestational age, male sex, lower birth weight, vaginal method of delivery, and lesser weight gain during hospital stay.

## Introduction

Non-invasive ventilation (NIV) is a way of administering mechanical respiratory assistance without the need for endotracheal intubation [1]. Out of the numerous available NIV modalities, continuous positive airway pressure (CPAP) and nasal intermittent positive pressure ventilation (NIPPV) are most routinely utilized [2]. CPAP enhances oxygenation by increasing functional residual capacity, decreasing airway resistance and effort of breathing, enhancing diaphragmatic function [3], and lowering upper airway resistance to minimize obstructive apnea [4,5]. CPAP includes undesirable consequences such as "CPAP belly syndrome" [6], nasal pain, and skin damage. In newborn facilities, CPAP is utilized both as the primary method of breathing and also following extubation from mechanical ventilation [7].

A considerable proportion of preterm newborns develop respiratory distress (RD) warranting breathing assistance due to low inspiratory effort, weak intercostal muscles, and poor diaphragmatic function. The duration of invasive mechanical ventilation (IMV) and supplementary oxygen increases the risk of bronchopulmonary dysplasia (BPD), intraventricular hemorrhage, and periventricular leucomalacia [8]. NIV has a lower risk of BPD as compared to IMV [9]. Using CPAP in the delivery room and NIPPV in the neonatal intensive care unit (NICU) minimizes the requirement for IMV in extremely preterm newborns [10]. NIPPV is preferable to CPAP since it minimizes the requirement for IMV in preterm babies with RD [2]. Early nasal CPAP (ENCPAP) has demonstrated a considerable risk of failure, notably in lower gestational ages, birth weights, and oxygenation parameters in the first hours of life [11-13]. Limited work is available reviewing NIV failure factors in gestational age 28-34 weeks in resource-limited poor countries. The present study was conducted to explore the link between the initial fraction of inspired oxygen (FiO_2_), time of surfactant administration, type of NIV support, and other parameters to enhance continuous respiratory support in the NICU in a resource-limited situation.

## Materials and methods

This was a longitudinal observational study undertaken in the intramural NICU at a tertiary care hospital in New Delhi from January 2021 to April 2022. The protocol was authorized by the institutional review board, followed by approval from the Institutional Ethics Committee on Human Research (IEC-HR) approval no. IECHR/2020/PG/46/60IECHR/2020/PG/46/60, dated 21/12/2020. Written informed consent was acquired from parents or legal guardians prior to research participation.

**Figure 1:**
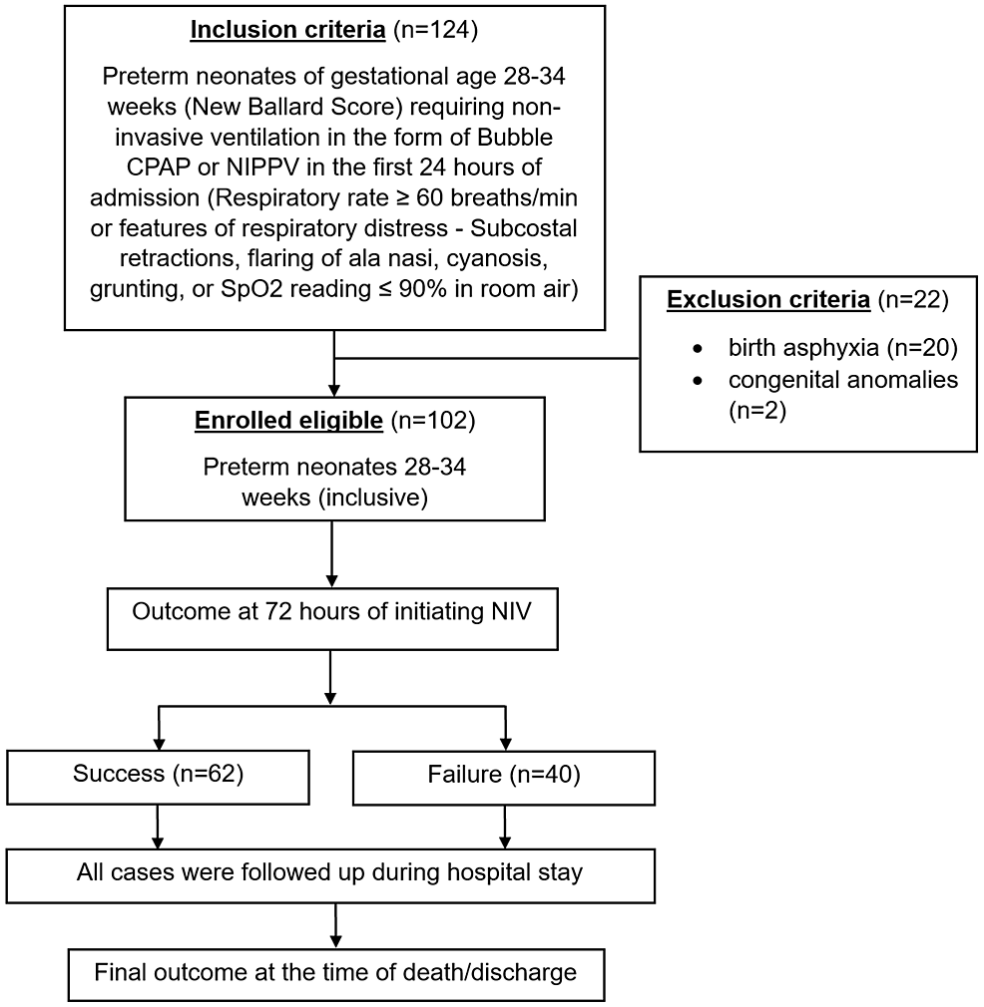
Flowchart of enrollment and evaluation. CPAP: continuous positive airway pressure, NIPPV: nasal intermittent positive pressure ventilation, NIV: non-invasive ventilation.

All preterm neonates admitted to the intramural NICU with a gestational age of 28-34 weeks and requiring NIV support during the first 24 hours of admission were eligible for inclusion in the trial. Patients with congenital abnormalities and birth asphyxia were excluded. One hundred two eligible newborns were enrolled, as indicated in Figure 1.

**Figure 2:**
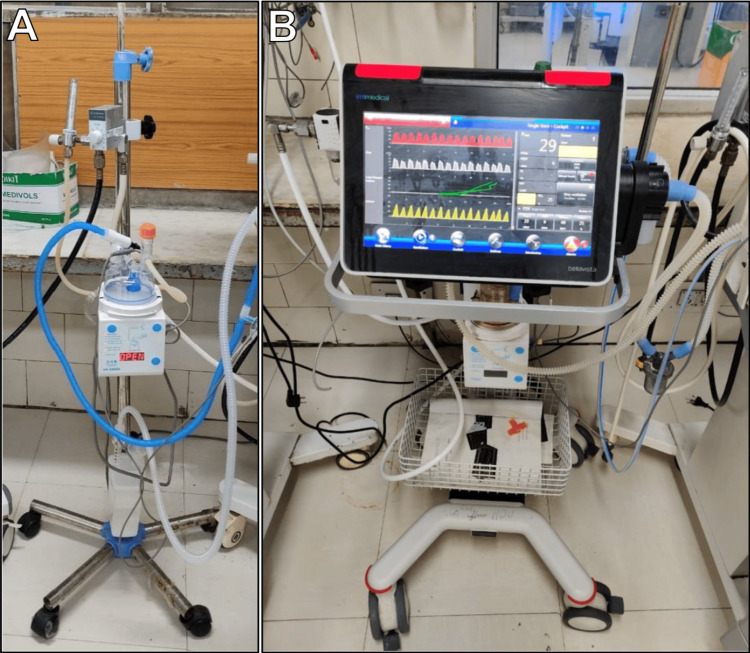
NIV machines. (A) Mediserv Bubble CPAP machine; (B) Bellavista ventilator. CPAP: continuous positive airway pressure; NIV: non-invasive ventilation.

Indications of NIV were based on the presence of ≥1 of the following: (i) respiratory rate ≥60 breaths/min; (ii) symptoms of RD: subcostal retractions, ala nasi flaring, cyanosis, grunting; or (iii) SpO_2_ values ≤90% in room air. NIV was delivered either in the form of CPAP using the Mediserv Bubble-CPAP machine via binasal prongs or NIPPV utilizing BellaVista ventilators via a binasal NeoRAM cannula, as indicated in Figure 2.

The demographic characteristics (birth weight, gestational age, maternal age) and clinical parameters, including vitals, systemic examination, and Silverman-Anderson score, were documented twice: first at the time of commencing NIV and again 72 hours after initiating NIV assistance. The gestational age computed using New Ballard scoring was regarded as the true gestational age. Surfactant Survanta® Beractant was used at a dose of 4 mL/kg and instilled using the InSuRe technique. We defined "post-surfactant stabilization" as the time taken following the instillation of surfactant to attain vitals and ventilatory settings equivalent to or better than pre-surfactant vitals and ventilatory settings. Outcome was assessed at 72 hours of commencing NIV in terms of 'NIV success” (no NIV or on NIV with positive end-expiratory pressure (PEEP)<8 cm H_2_O and FiO_2_<0.7) or “NIV failure” (NIV with PEEP≥8 cm H_2_O or FiO_2_≥0.7, or intubation or death). All recruited cases were tracked throughout the hospital stay and examined during discharge or death for a final diagnosis, the development of bronchopulmonary dysplasia (BPD), and weight gained or lost.

Sample size

The sample size was determined on the basis of research by Rocha et al [14]. Parameters like the initial FiO_2_ level, surfactant administration, hours of life at the first dose of surfactant, and presence of respiratory distress syndrome (RDS) in NIV failure and success groups were considered in the sample size calculation, and using the “2 Independent Sample Hypothesis Testing Formula” to estimate the difference at alpha error 0.05 and power = 80%, a sample size of 50, 20, 20, and 45 in each group was required, respectively. Considering the success rate as 50% in our NICU, we took our sample size as 50 cases in each group, i.e., the sample size was 100.

Statistical analysis

Data was imported into a Microsoft Excel sheet (Microsoft Corp., New York, USA) and analyzed using IBM SPSS Statistics for Windows, Version 20 (Released 2011; IBM Corp., Armonk, New York). Continuous data were reported as medians and interquartile ranges and compared using a non-parametric Mann-Whitney U test. Categorical data were reported as proportions and compared using the Chi-square test. P<0.05 was deemed significant.

## Results

Out of 102 participants enrolled, 61.8% (n = 63) were males and 38.2% (n = 39) were females, with a median (IQR) gestational age of 31 (29-33) weeks, a median maternal age (IQR) of 26 (23.75-29) years, a median (IQR) birth weight of 1217 (1053-1484.5) grams, 83.3% (n = 85) singleton deliveries, and 17 (16.7%) twin deliveries; neonates born to multi-parous mothers comprising 66.7% (n = 68) and primi-parous mothers 33.3% (n = 34); neonates born out of lower segment cesarean section (LSCS) comprising 51% (n = 52) and vaginal delivery 49% (n = 50); neonates born to unbooked pregnancy comprised 83.3% (n = 65) and to booked pregnancy 16.7% (n = 37); neonates initially put on CPAP comprised 71.6% (n = 73) and on NIPPV 28.4% (n = 29).

**Figure 3:**
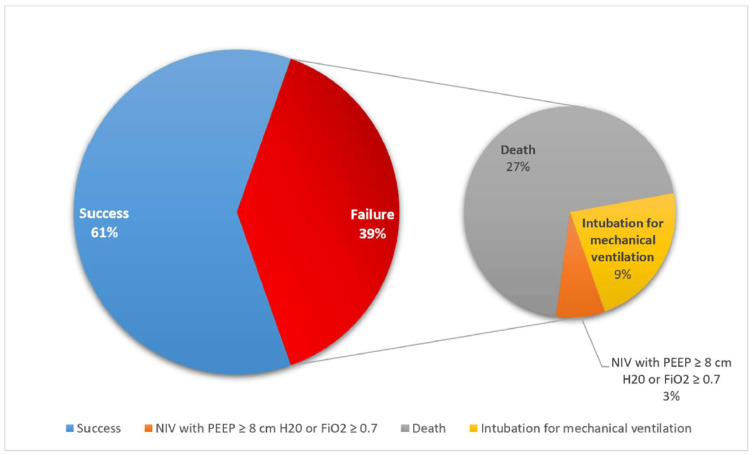
NIV status at 72 hours of NIV and causes of failure. NIV: non-invasive ventilation, PEEP: positive end-expiratory pressure, FiO_2_: fraction of inspired oxygen.

Table 1 shows demographic, clinical characteristics and ventilatory parameters, and their associations with NIV failure and NIV success. Out of 102 patients, 40 (39%) experienced NIV failure at 72 hours of initiating NIV, which was constituted by death (27%), intubation for IMV (9%), and NIV with PEEP ≥8 cm H_2_O or FiO_2_≥0.7-3%, as shown in Figure 3. Out of 40 NIV failures, after excluding 28 fatalities (cause of NIV failure), of the remaining 12 individuals, 75% died and 25% survived. NIV failure was associated with higher fatalities (p<0.001). Males represented 75% of NIV failures and 53.2% of the NIV success group (P = 0.027).

**Table 1:** Association of various demographic parameters, clinical and ventilatory parameters with NIV failure and NIV success. **P<0.05 is significant. AGA: appropriate for gestational age, CPAP: continuous positive airway pressure, FiO_2_: fraction of inspired oxygen, HOL: hours of life, IQR: interquartile range, RDS: respiratory distress syndrome, NIV: non-invasive ventilation.

Factor	Total (n = 102)	NIV failure (n = 40)	NIV success (n = 62)	P-value
Median maternal age (IQR)	26 (23.75–29)	26 (23–29)	27 (27–29.5)	0.623
Median gestational age (IQR)	31 (29–33)	29 (29–31)	31 (29–33)	0.015**
Median birth weight (IQR)	1217 (1053–1484.5)	1087.5 (960–1293.5)	1333 (1100–1565)	0.003**
Median weight gained/lost (IQR)	16 (-50 to 86)	-21 (-70.5 to 11.75)	53 (-28.25 to 111.5)	<0.001**
Gravida–primi n (%)	34 (33.3)	17 (42.5)	17 (27.4)	0.115
Vaginal mode of delivery, n (%)	50 (49)	27 (67.5)	23 (37.1)	0.003**
Singleton, n (%)	85 (83.3)	31 (77.5)	54 (87.1)	0.204
Gender-Male, n (%)	63 (61.8)	30 (75)	33 (53.2)	0.027**
Booked pregnancy, n (%)	37 (16.7)	14 (35)	23 (37.1)	0.83
AGA, n (%)	96 (94.1)	37 (92.5)	59 (95.2)	0.577
Median initial FiO_2_ (IQR)	50 (40–50)	50 (50-50)	50 (40–50)	0.069
Presence of RDS, n (%)	52 (51)	18 (45)	34 (54.8)	0.332
Antenatal steroids, n (%)	51 (50)	15 (37.5)	36 (58.1)	0.127
Surfactant administration, n (%)	24 (23.5)	13 (32.5)	11 (17.7)	0.086
Surfactant dose1 HOL (IQR)	2.25 (2–4.75)	2 (2-6)	2 (2–5)	0.766
Post-surfactant stabilization in minutes (IQR)	15 (10–25)	14 (10–25)	14 (10–25)	0.975
Initial mode CPAP, n (%)	73 (71.6)	22 (55)	51 (82.3)	0.003**

The NIV failure group had a shorter median gestational age (IQR) of 29 (29-31) weeks compared to 31 (29-33) weeks in the NIV success group (P = 0.017). Children delivered via vaginal birth included more of the NIV failure group (67.5%) than 37.1% of the success group (P = 0.003). The median birth weight (IQR) was 1088 (960-1293.5) grams in the NIV failure group, which was smaller than 1333 (1100-1565) grams in the NIV success group (P = 0.003). The median gained or lost weight (IQR) was a loss of 21 (−70.5 to 11.75) grams in the NIV failure group and a gain of 53 (−28.25 to 11.5) grams in the NIV success group (P<0.001).

Out of newborns initially receiving CPAP, 30.1% had NIV failure, whereas out of neonates initially receiving NIPPV, 62.1% had NIV failure. NIPPV had a greater NIV failure rate than CPAP as the initial mode of breathing (P = 0.003). NIV failure was not impacted by maternal age, gravida status, singleton/twin pregnancy, antenatal steroids, initial FiO_2_, surfactant administration, post-surfactant stabilization, or RDS presence. As just one neonate developed BPD, enough data were not available for statistical evaluation (Table 2).

**Table 2:** Association of final outcome with NIV failure and NIV success. **P<0.05 is significant. NIV: non-invasive ventilation.

Results		Status at 72 hours		Total	P-value
		NIV failure	NIV success		
Final outcome	Survived	3	53	56	<0.001
	Death	37	9	46	
Total		40	62	102	

## Discussion

This study was undertaken with the purpose of establishing the parameters related to the failure of NIV in preterm neonates of 28-34 weeks gestation in resource-limited settings. In our analysis, NIV failure showed a significant association with a lower median gestational age, male gender, a lower median birth weight, vaginal delivery, and a lower median weight gain or loss.

In a study done by Rocha et al. [14] in 2013 in 131 preterm neonates of 26-30 weeks gestation, with a median gestational age (IQR) of 29 (25-29) weeks, males (comprising 66.6% of the failure group) experienced higher early nasal CPAP (ENCPAP) failure, but gestational age was comparable in the failure and success groups. Another study by De Jaegere et al. [13] in 2012, done in 182 preterms less than 30 weeks gestation (mean gestational age (SD) of 27.9 (1.5) weeks), revealed that male gender (comprising 64.5% of the failure group), lesser gestational age, and lesser birth weight ≤800 gm were associated with ENCPAP failure. Ammari et al. [11] in 2005 reported that CPAP failure was associated with a lower gestational age and a smaller birth weight. This was comparable to our research finding, where in the NIV failure and success groups, males constituted 75% and 53.2%, respectively (P = 0.027). However, our study was done at a somewhat higher median gestational age (IQR) of 31 (29-33) weeks, with the median gestational age (IQR) in the success and failure groups being 31 (29-33) weeks and 29 (29-31) weeks, respectively (P = 0.017). Also, lower birth weight was strongly linked with NIV failure (P = 0.005). Contrary to this, no significant statistical connection was established by Rocha et al. between ENCPAP failure and birth weight. The aforementioned outcomes of our study can be attributed to the immaturity of the lungs and other organs, a greater risk of infection, and more intense ventilatory assistance, such as invasive ventilation, necessary in very low birth weight (VLBW) and extremely low birth weight (ELBW) newborns.

Rocha et al. [14] also observed that the factors connected to ENCPAP failure in preterm babies were the requirement for resuscitation with FiO_2_>0.30 or the need for FiO_2_≥0.40 in the first four hours of life. De Jaegere et al. (2012) concluded that individuals who required FiO_2_>0.25 at one and two hours of age were related to early nasal CPAP (NCPAP) failure [13]. In another study in 2013 by Dargaville et al., CPAP failure was shown to be predicted by FiO_2_>0.30 in the first hours after delivery in neonates on CPAP [15]. However, in our study, the primary aim of proving a relationship between initial FiO_2_ and NIV failure was not attained because of a smaller sample size and partly because NICU residents had an innate inclination to start ventilating with a high median initial FiO_2_ of 50%.

Rocha et al. [14] likewise showed that RDS formed 68.7% of the failure group and 63.4% of the success group (P = 0.018). Ammari et al. [11] in 2005 similarly observed that CPAP failure was substantially related to severe RDS. No such link could be shown in our investigation. This could be attributed to differences in gestational age among the studies, with our study done at a slightly higher median gestational age of 31 weeks (29-33), where RDS is less prevalent morbidity and a lesser contributory factor to NIV failure as compared to other studies done in more premature neonates.

Rocha et al. [14] also detected a substantial relationship between ENCPAP failure and surfactant treatment. Surfactant was delivered in 77.7% of the failure group and 27.8% of the success group (P<0.0001), and the median HOL at the first dose of surfactant administration (IQR) was 10.5 (2-26) hours in the failure group (P = 0.023). Opposed to this, in our investigation, surfactant administration encompassed 32.5% of the failure group (P = 0.086), and the median HOL at the first dose of surfactant administration (IQR) was 2 (2-6) hours in the failure group (P = 0.766). This divergence across studies may be attributable to the greater gestational age of our research participants, where RDS is a less prevalent illness, and consequently, the requirement for surfactant is minimized, distinct from previous studies done in more preterm newborns. Also, we had a more active strategy and early delivery of surfactant as compared to another research.

Early NIPPV was superior to NCPAP in preventing NIV failure, according to a Cochrane study published in 2016 [16]. However, comprehensive research done in 2013 in 1009 children with a birth weight of less than 1000 grams and a gestational age of under 30 weeks by Kirpalani et al. [17] revealed no difference in neonatal outcomes with NIPPV versus NCPAP in terms of post-extubation and primary support. In recent research in 2016, Oncel et al. [18] reported that while NIPPV greatly decreased the requirement for IMV during the first 72 hours of life, there was no significant difference in NIV failure between NCPAP and NIPPV in children born at fewer than 30 weeks of gestation. Opposed to the abovementioned studies, CPAP performed better as an initial mode of respiratory support than NIPPV in the present study (P = 0.003). This can be attributable to numerous things. CPAP machines were widely available compared to ventilators necessary for NIPPV in our resource-limited scenario. A higher NIV failure threshold of FiO_2_≥0.7 and PEEP≥8 cmH_2_O were used in our NICU, owing to the high patient turnover rate and limited infrastructure, allowing more patients to be managed on NIV, as ventilator availability is a challenge in a resource-limited setting like ours, and in turn, lessening the burden of the IMV requirement. More ill neonates were directly put on NIPPV, thereby increasing the NIPPV failure rate as the risk of being invasively ventilated later increases. As per standard recommendations from European Consensus Guidelines 2019 [19], the cutoff for selecting therapy is FiO_2_>0.30 on a CPAP pressure of at least 6 cmH_2_O. Similar cutoffs are being tried to be applied in our NICU, with new procedures ordering residents to start ventilation at a reduced FiO_2_ of 0.3 or 0.4.

In a study by Buyuktiryaki et al., multivariable logistic regression analysis demonstrated that antenatal steroid administration (OR: 0.49, 95% CI: 0.27-0.90; P = 0.02) and gestational age <28 weeks (OR: 2.03, 95% CI: 1.18-3.49; p = 0.01) were independent factors that influence the failure of NIV within the first 72 hours of life [20]. However, in our investigation, we discovered that antenatal steroid treatment had no influence in avoiding NIV failure (P = 0.127), which can be related to the larger gestational age of our study participants, rendering RDS a less prevalent illness.

Out of the entire 102 participants, 45.1% (n = 46) died, which includes 28 fatalities as a result of NIV failure, as indicated in Table 2. The remaining 18 fatalities included eight participants in the failure group. This significant fatality rate can be linked to the lesser gestational age of our participants, inadequate resources, and significant patient turnover. Also, beyond 72 hours, numerous other complicating variables, such as sepsis and other consequences of prematurity, lead to mortality and can't be blamed on NIV failure alone.

In our study, the individuals were enrolled using the correct gestational age by New Ballard Scoring to ensure comparable baseline data before continuing further. Observing the ongoing activities and the subsequent outcomes helps us discover the bottlenecks in our approach and management, in turn helping us to understand the predictors of NIV failure and develop robust protocols to prevent the same.

Limitations

Significant associations couldn’t be established between the majority of our research parameters, warranting a future investigation with a larger sample size. Our primary aim of demonstrating a relationship between initial FiO_2_ and NIV failure could not be achieved because, in the NICU, there is a natural propensity for the residents to start ventilating with a high initial FiO_2_. Another arm for measuring FiO_2_ supplied by indigenous CPAP, which comprises a substantial proportion of ventilatory support offered in our intramural NICU, couldn’t be tested and had to be eliminated from our study owing to a shortage of instruments to monitor FiO_2_ delivered by such devices. We also couldn’t assess the link between BPD and NIV failure, as just one patient developed BPD.

## Conclusions

The goal of this study was to investigate the parameters related to the failure of non-invasive ventilation in preterm neonates in gestation 28-34 weeks. Significant statistical associations were established with lesser gestational age, male gender, lesser birth weight, mode of delivery, shorter median hospital stay, and lesser average weight gain, while NIV failure was not affected by maternal age, gravida status, mode of delivery, singleton or twin pregnancy, use of antenatal steroids, initial FiO_2_, surfactant administration, time required for post-surfactant stabilization, or presence of RDS. Enough data were not available to demonstrate an association between NIV failure and BPD. In our study, CPAP performed better as an initial technique of respiratory support than NIPPV, contrary to numerous earlier studies. Higher FiO_2_ and pressure cutoffs were utilized to define NIV failure in order to manage more patients on NIV for a longer duration and, in turn, minimize the burden of invasive mechanical ventilation and intubation. The outcomes of this study have brought about a shift, implementing updated standards that prefer lower initial FiO_2_ settings when initiating NIV assistance in our NICU.
